# Progress of Medical Science

**Published:** 1857-08

**Authors:** 


### Progress of Medical Science.

Prof. F. S. Smith, in the July number of the North American Medico-Chirugical Review, gives the result of experiments upon digestion which corroborates the conclusions derived from previous experiments, upon Alexis St. Martin, viz: "that the acid reaction" of the gastric juice "is due to lactic acid; that the nitrogenized food is converted into albuminose; that fatty articles undergo no change beyond minute division; that amylaceous or starchy materials (in man) are converted into glucose or grape sugar."

The latter of these conclusions having been objected to, Prof. S. secured the aid and services of Dr. Brown-Sequard, who has the power of emesis at will, for the purpose of further experiments, which, so far as they went, tend to establish the truth of the conclusion objected to.

In the same number of the above journal, Dr. Levergood, of Wrightsville, Pa., relates a series of three cases in the practice of one of his professional friends, in which puerperal fever was clearly shown to have originated from erysipelas, a case of which the atttending physician in several obstetric cases was in attendance.

---

It is well known that for some time it has been a question of debate whether diabetes mellitus does not always depend upon certain pathological conditions of the great nervous centres. Some of the conclusions from the researches of M. Bernard have seemed to render this probable. We remember the argument (if it may be so called,) in support of this position, as advanced some years since, (1850,) by Dr. J. Adams Allen, formerly Prof. of Physiology in Michigan University, upon the witness stand, in an interesting trial of a man for the murder of his son. The prisoner had for some years suffered from the above disease and for some months had evinced undoubted signs of mental aberration. Although wealthy, he claimed that his sons were about to starve for want of bread—indeed, he asserted that the whole race was about to be destroyed by starvation, as there was no more flour, although he was himself a produce dealer, with hundreds of barrels of flour on hand, and was daily engaged in buying and selling large quantities of the same. To prevent so terrible a death to his children and himself, he killed the one and attempted to enter the house of the other with a like murderous purpose, and failing therein, essayed his own death by drowning, but was rescued.

Dr. Allen having examined the prisoner, and indeed attended him for some time while in prison, was called upon as a medical witness. In his opinion the prisoner was a subject of monomania, and he gave it as his farther opinion, from examinations and the history of the case, that the insanity and the diabetic disease were the results of one and the same cause—cerebral disease.* We have no space to give the full testimony in the case, but refer to it now because reminded of it as corroborative of an interesting paper presented to the French Academy of Sciences, in March last, by M. Leudet. We copy the cases cited by the author, as found in brief in the Virginia Medical Journal:

CASE I.—Woman of 32 years, afflicted at the sixth month of pregnancy, with loss of vision of the left eye, without any paralytic phenomena in the limbs. There was headache and vomiting. Seven and a half months subsequently, a sudden invasion of the comatose symptoms, which gradually subsided after twenty-four hours. There was now discovered palsy of the 3d and 5th cranial nerves on the left side, slight softening of the left cornea, anæsthesia of the left half of the face, nasal mucous membrane, and tongue. Intense thirst and the general signs of diabetes supervened. The presence of sugar in the urine was demonstrated by liquor potassæ and the Barreswil solution. Iodide of potassium was administered, and all the symptoms were relieved, except the corneitis, which terminated in the loss of the humors of the eye. (Observed at Hotel Dieu of Rouen.)

CASE II.—Woman of 53 years, suddenly affected by right hemiplegia of cerebral origin; epileptic fits occurring for a short period; two years subsequently, well marked diabetes; a year afterwards, albuminuria and a state of cachexia. (Observed in M. Rayer's wards at La Charite.)

CASE III.—Woman of 80 years, sudden attack of left hemiplegia; eighteen months afterwards, increased thirst, sugar in the urine; gangrene of the right foot; death. (Rouen Hospital.)

CASE IV.—Woman of 39 years, affected, in the sixth month of gestation, with paraplegia and convulsions; gradual disappearance of symptoms, persistence only in vertigo; six years subsequently, hæmorrhages; then dyspeptic symptoms; then diabetes mellitus; intercurrent variola; death. (Rayer's Service.)

M. Macario administered lilac leaves as a febrifuge, in 20 cases, some of which had been unsuccessfully treated by qui-

* The prisoner died in an insane hospital about fifteen months after the trial, having experienced no amelioration of the cerebral or diabetic disease.

nine and arsenic, with entire success in 15 cases of the 20. A decotion of the leaves was given while the stomach was empty.

---

Dr. Schubert (Med. Zeitung,) treats itch successfully with a solution of eight ounces of soft soap and four ounces of common salt in a quart of water. The patient is to be well rubbed with the solution twice daily, and a cure follows in three or four days.

---

Dr. Daniel Ayers, in the New York Journal of Medicine, reports a case of dislocation of the cervical vertebra, which was reduced on the tenth day after the accident, the patient having suffered no permanent injury therefrom.

---

Veratrum Viride has been used successfully in the treatment of aneurism, as proposed by M. Fergusson, by Dr. Geo. C. Blackman, Prof. of Surgery in the Medical College of Ohio. The patient was put upon the tinct., six drops every 3 hours, April 11. On the 12th the pulse was reduced from 94 to 65, when, before the class, Prof. B. attempted to discharge a part of the fibrinous layers of the aneurismal sac (of the *femoral,*) by forcible pressure into it with the thumbs. The pulse coming up within a couple of hours thereafter to 100, full and strong, he was bled oz.ix, with the effect of reducing it to 50. Skey's tourniquet was applied with moderate force between aneurism and Pouport's ligament until the 17th. Pulsation entirely wanting on the 22d, and on the 30th patient complains only of weakness. Discharged, May 21, cured.

Iowa Med Journal

---

Iodoform is highly recommended as an eligible preparation when it is desirable to administer iodine or its compounds in large quantities. It is well borne by the stomach, and yet promptly produces all the constitutional and alterative effects of other preparations of iodine.

---

*Bony Union after Fracture of the Cervix Femoris within the Capsule.*—T. Bryant, Esq., exhibited to the Pathological Society of London (Feb. 3, 1857) a specimen of this, which is worthy of note in connection with the valuable paper of Prof. Mussey, inserted in our preceding number.

"Mary H., aged 60, a lunatic inmate of the asylum at Guy's Hospital five years ago, when walking in the garden, fell and fractured her right thigh bone. All the symptoms of fracture of the neck of the femur within the capsule were present, clearly indicating the character of the injury. A long splint was applied; but much difficulty was experienced in preserving the leg in the right position from the restlessness of the patient. After some weeks' confinement she was allowed to sit up, but her health soon began to fail, and she never walked again, and on June 30 she died. The specimens shown consisted of the upper parts of both thigh bones. On the injured side union was complete, and had partly been effected by bone, partly by cartilage, and in part by fibrous tissue. The whole of the neck had been absorbed, and the articular head was united directly to the base of the great trochanter. The union was firm, and the head of the bone was much indurated. [British Med. Jour., March 14.

---

*Ligature of Arteries in Suppurating Wounds.*—In one of his recent clinical lectures, M. Nelaton made the following observations, the occasion being a secondary hemorrhage in the palm of the hand. Nothing is more difficult, he observed, than to arrest a hemorrhage of the hand, especially when this is consecutive—that is, when the wound is covered by pyogenic granulations. If not previously instructed as to the proper management of these secondary hemorrhages, you will be extremely embarrassed. The blood flows, you employ compression and it ceases; but the hemorrhage will not be long before it returns, and will then be uninfluenced by compression. If compression be made above the wound, œdema takes place in all the subjacent parts, and the hemorrhage soon returns. The radial, or the ulnar, or the brachial may be tied, and yet the bleeding does not stop. Meeting such a case, M. Nelaton formerly was quite at a loss to know what to do, impressed as he was with Dupuytren's *dictum*, that arteries in the suppurating wound will not bear the ligature, the premature fall of this infallibly giving rise to a return of the hemorrhage. Nevertheless he ventured to tie the two ends of the bleeding vessel of the palmar arch; and although the ligature fell sooner than usual, no hemorrhage followed. He has since then frequently tied vessels under analogous circumstances, and has never seen hemorrhage as a result of the fall of the ligature. Although, therefore, this fall takes place earlier (usually about the third or fourth day) then is the case with a ligature applied to a healthy artery, it is not premature, for the bleeding does not follow.

Examining the matter experimentally upon the dead body, M. Nelaton has found that ligatures applied to arteries in a state of suppuration (as in patients who have died after amputation) produce identically the same effects upon the coats of these vessels as upon arteries remote from the seat of inflammation: the same division of the inner coats, and preservation of the outer, taking place in the two cases. He feels, therefore, perfect confidence in the soundness of the practice, supported as it is by numerous cases that have oocurred to him, both in private and hospital practice. [B. and F. Med.-Ghir. Rev., April, 1857, from Gaz. des Hopitaux, 1857.

---

*Liquor Sodæ Chlorinatæ as a Local Application in Smallpox.*—Mr. John Gabb states (Am. Jour. of Med. Sci., July, 1857, from British Med. Jour., April 4, 1857,) that he has found a weak solution of the liquor sodæ chlorinatæ highly beneficial in the affection of the mouth and throat in cases of smallpox. In each case in which he has employed it the effect has been extraordinary; one washing of the mouth and gargling of the throat has restored the patient to comfort and ability to speak or swallow without difficulty. The strength he has used has been a drachm to a half pint of water. Applied to the skin, it has had the effect to allay the troublesome itching; and he thinks it not unlikely that a much stronger solution, applied in the earlier stage of the eruption, might be as efficient in preventing pitting as some other remedies which have been recommended, whilst, at the same time, it could be more easily used.

Finding the solution so useful in allaying the itching of smallpox, he gave an old woman, aged 86, who had been for more than twelve months tormented with pruritus, and had tried various remedies without effect, the same to use as a lotion. In a few days she came for more, and said she had never used anything that gave her so much relief.

---

*Digestion and Absorption of Fatty Substances without the Concurrence of Pancreatic Juice.*—In July, 1856, M. Colin, of Alfort, read to the Academy of Medicine a memoir on this subject. At a meeting of the Academy on the 21st of April last, M. Bernard made an interesting report on this memoir. We have room only for the following conclusion which he presents:

"Since in animals of the bovine species, three or even four days after the excretory duct of the pancreas has been tied, and the pancreatic juice is made to flow out of the economy, there may be obtained from the thoracic duct, in 24 hours, more than 40 litres of perfectly emulsioned chyle, from which may be extracted, by ether, a notable quantity of fat; the pancreatic juice in these animals is not necessary either for the absorption of fatty bodies or for the formation of the emulsioned chyle."

M. B. says *necessary* and not *useful*, and his conclusions refer only to animals of the bovine species, though analogy would support him, he states in further generalizing. [Moniteur des Hopitaux, April 25, 1857.

---

Dr. Liverey, in the Boston Medical and Surgical Journal, recommends the root of the *pœonia officinalis* as an efficacious remedy in the convulsions of children. "The mode of administration is this:—Take of the dried root, grated, half a teaspoonful; scald it, sweeten, and give the whole at once to a child three to five years old *ter die.* To an infant the same amount may be given in divided doses during the day or 24 hours.".

blistering

---

M. Thierry states that he treats hæmorrhoids, even when large, by first liberating them and then applying the perchloride of iron to the denuded surface, under the influence of which they sink and disappear. The cure may not be radical and they may re-appear under the influence of causes that originally produced them; but this is only the case after a considerable period, and in the meantime health is restored and occupation resumed. [Ohio Med. Surg. Jour., from Union Med.

---

The perchloride of iron is regarded as a valuable agent in the treatment of some forms of nævus by English surgeons. Small quantities are repeatedly injected with the effect of producing coagulation of the blood in the tumor and without the supervention of sloughing. When the nævus is small, Mr. Bowman introduces the perchloride by means of a ligature of silk which is saturated with the remedy, using a broad needle for the purpose, and first puncturing the skin with a lancet to prevent the liquid from being squeezed out in entering the skin. The same remedy has been recommended by Thierry for treatment of varix.

### Proceedings of Iowa State Medical Society.

The State Medical Society met at Iowa City, on Wednesday, the 10th of June, at 2 o'clock, P. M., and a temporary organization was made by the appointment of Dr. Stone, of Iowe City, President *pro tem*, and Dr. Fountain, of Davenport, Secretary, in the absence of the proper officers of the society.

In addition to the delegates in attendance, the following persons were elected members, viz:

Drs. E. S. Barrows, T. S. Saunders, J. W. H. Baker, H. Carpenter, of Davenport; J. J. Saunders, J. H. Boucher, J. H. Ealy, of Iowa City; H. Bunce, of Toledo; J. Gamble, of Le Claire.

The following committee was appointed on the nomination of permanent officers of the society, for the ensuirg year, viz:

Drs. Baker, Barrows, J. J. Saunders, Siveter, and Ealy. Adjourned till to-morrow at 10 o'clock, A. M.

---

THURSDAY, 10 o'clock, A. M.

The minutes of yesterday were approved. The committee on the nomination of officers reported, and their report was received, as follows:

Dr. THOS. SIVETER, President,
" M. B. COCHRAN, Vice President,
" J. C. STONE, Secretary,
" T. J. Saunders, Cor. Secretary,
" JAMES GAMBLE, Treasurer,
" H. CARPENTER, Librarian.

Dr. — Bird, of Mt. Pleasant, — Reeder, of Muscatine, J. H. Boucher, J. H. Ealy and J. J. Saunders, of Iowa City, and E. J. Saunders, of Davenport—Censors.

Pending the above nominations, the society proceeded to the election of the following new members, viz:—Drs. H. Murray, T. S. Mahan, Wm. Vogt, M. Chchran, F. Lloyd, E. J. B. Statler and Wm. Carus, all of Iowa City.

The foregoing nomination of officers for the ensuing year was than taken up and they were respectively elected.

The President on being conducted to the chair by the retiring officers pro tem, remarked:

"It may be expected that on taking the chair, something should be said befitting the occasion. But I have to express my regret at so few members being present. Yet I trust, from the character of those now here, that a nucleus may be formed for larger gatherings hereafter; that we may go out of the lethargic position in which we seem to be; consult the credit and promote the honor of the profession, and build up a good and efficient society, that may be advantageous to the community and an honor of the state."

On the nomination of Dr. Knox, of Iowa City, for membership, by Dr. Vogt, there arose some discussion.

Dr. Cochran said—I regret the nomination, as I feel bound to oppose it. Dr. K. has been rejected a membership in our County society, for consulting with irregular physicians, and encouraging quacks.

Dr. Vogt said—That was my reason for nominating him here, that he might come before another tribunal. He is a member of the American Medical Society of New York, and has all the requisites of the rules of this Society.

Dr. Cochran—The American Medical Society is not located in New York; and if they knew Dr. Knox's course here, they would exclude him.

Dr. Fountain—I am somewhat acquainted with the case of Dr. Knox, and I think it not a good precedent to elect a member who has been rejected by the County society. I wish the gentleman would withdraw the nomination.

Dr. Vogt signified his willingness to do so, and accordingly, Dr. Knox's name was withdrawn.

Dr. M. J. Morseman, of Iowa City, was then elected.

The following committee was appointed to revise the Constitution and By-Laws of the society, and to report next year, viz., Drs Gamble, Stone and Cochran.

The standing committees of last year, after some remarks by the President and others, were, on motion of Dr. Murray, all discharged, and Messrs. Gamble, Burrows and Boucher, were appointed a committee to nominate standing committees on medical subjects for the next year.

The nomination of delegates to the American Medical Society was also referred to the foregoing committee.

The Treasurer for the last year, Dr. Siveter, reported receipts $93; expenditures $4 75; balance in hand$88 25. Report adopted with the thanks of the society for his fidelity.

Dr. Ealy moved that Mt. Pleasant be the place of the next meeting; for which motion Dr. Murray moved to substitute Davenport, which substitute was lost; and the original motion was carried.

Considerable discussion here arose on the subject of past dues from the Treasury, the absence of the officers and members in the south part of the state, and the independence of this society with respect to any particular medical schools. On motion of Dr. Carpenter to appoint the secretary a publishing committee, with authority to draw on the treasurer for the means to print the proceedings in a panphlet; which motion was amended by Dr. Fountain so as to include the treasurer in the committee on publication, was carried.

Dr. Boucher. of the committee to nominate standing committees on medical topics, reported, and their report, as amended, was adopted, as follows:

On Physiology, Dr. Huey, of Fairfield; Surgery, Dr. Stone of Iowa City; Obstetrics, Dr. Fountain, of Davenport; Pathology, Dr. M'Gugin, of Keokuk; Pulmonary Consumption, Dr. Cochran, of Iowa City; Cutaneous Diseases, Dr. Vogt, of Iowa City; Quackery, Dr. Rauch, of Burlington.

The committee further reported the following delegates to the American Medical Society, to wit:—Drs. Wood, of Ottumwa; Murray and Boucher, of Iowa City; Gamble, of Le Claire; Cousins, of Albia; Burrows and Fountain, of Davenport; Cleaver, of Columbus City; and Reeder, of Muscatine. The delegates were authorized to appoint substitutes.

Dr. Cochran offered the following resolutions:

*Resolved,* That the delinquency on the part of the secretary of this society, elected last year, in not giving notice of the meeting of the State Medical Society, as required by the constitution, and in not furnishing the minutes of the last year's meeting; and of the committee on publication for not attending to the duties of their office, is deserving the severest censure of this body; and that the secretary of this body be requested to obtain the Records of the society and place them in the hands of the committee on publication, as soon as practicable.

*Resolved,* that a copy of these proceedings be sent to the secretary of last year, and to each of the members of the committee on publication.

The President suggested, as to the resolution, that if there were any lesion in the body, the wound might be made larger by such a censure upon delinquents, which might reflect upon the honor of the whole body. He hoped it would be considered before the resolution passed.

Dr. Murray thought if the wound should be enlarged a healthy granulation would take place, and the body be healed.

The President rejoined that the committee on publication of last year had done better than the one of the year before. They published the proceedings in a pamphlet form, and, in that respect they were not so delinquent.

Dr. Cochran replied that this was the first time that he had heard of the publication, and he supposed it was the duty of the committee to distribute the proceedings also. If the previous committee were thus delinquent, then such a resolution ought to have been passed a year ago.

Dr. Murray regretted the necessity for passing such a resolution, but hoped it would be understood hereafter, that officers were expected to discharge their duty, or receive censure, and in case of fidelity have a vote of thanks. The resolutions were adopted.

On motion of Dr. Murray, the address of Dr. Cochran, prepared for this occasion, was deferred to the next annual meeting.

At the suggestion of the President, the remainder of the session was spent in the familiar statement of matters of professional science and practice, which was entered into with considerable spirit and interest.

In reply to a query of Dr. Vogt, whether special remedies were not in fact mere empyricisms, and next door to quackery, the President said:

Like an old man and a young one in a boat, with a line and lead, it was desirable to sound as they go; so, by degrees, the young practitioner would get experience and practice.

On motion of Dr. Gamble, a vote of thanks was tendered to the physicians of Iowa City, for their kindness, attention and hospitality; and on motion of Dr. Murray, also to Rev. S. Storrs Howe, for voluntarily reporting the proceedings of the society.

Thereupon the society adjourned to meet at MT. PLEASANT, on the second Wednesday in June next.

After the adjournment, the members of the society repaired to the Clinton House, and partook of a sumptuous repast, prepared at the expense of the Iowa City Medical Society, discussing the rich entertainment, provided by Messrs. Troxel and Wentz, till the hour of departure by the cars called away the guests from abroad.

The only regret manifested was that there were not more persons present from a distance to share the festivities of the occasion.

