# Errata

**Published:** 1857-08

**Authors:** 


### ERRATA.

On page 144, third article from top, second line, fourth word, read —"blistering," instead of "liberating,"—thus: "by first blistering them," &c.

